# Shall We Change the ʒ Mark?

**Published:** 1888-01

**Authors:** 


### SHALL WE CHANGE THE ʒ MARK?

Dr. P. H. Adams, of Florenee, Texas, very sensibly suggests that we change the ʒ symbol to the letter (D) or the initial letter in the word *Drachma*—a drachm. This is a good suggestion, but will not at once be understood. The change is important in order to avoid mistaking the drachm for the ounce mark, which frequently occurs.

We suggest that a resolution be adopted by every State Medical Association at the spring meetings, recommending the change, and that every medical journal refer editorially to the fact in advance of said meetings, so as to put the State societies and the entire profession on notice of the proposed change.

